# Review on the roles of specific cell-derived exosomes in Alzheimer's disease

**DOI:** 10.3389/fnins.2022.936760

**Published:** 2022-07-28

**Authors:** Yutong Zou, Danni Mu, Xiaoli Ma, Danchen Wang, Jian Zhong, Jing Gao, Songlin Yu, Ling Qiu

**Affiliations:** ^1^Department of Laboratory Medicine, Peking Union Medical College Hospital, Peking Union Medical College & Chinese Academy of Medical Science, Beijing, China; ^2^Medical Science Research Center (MRC), Peking Union Medical College Hospital, Peking Union Medical College and Chinese Academy of Medical Science, Beijing, China; ^3^Department of Neurology, Peking Union Medical College Hospital, Peking Union Medical College & Chinese Academy of Medical Sciences, Beijing, China; ^4^State Key Laboratory of Complex Severe and Rare Diseases, Peking Union Medical College Hospital, Peking Union Medical College & Chinese Academy of Medical Sciences, Beijing, China

**Keywords:** Alzheimer's disease, mild cognitive impairment, exosome, biomarkers, neuron, glial cells

## Abstract

Alzheimer's disease (AD) is the sixth leading cause of death worldwide and cannot be effectively cured or prevented; thus, early diagnosis, and intervention are important. The importance of exosomes, membrane-bound extracellular vesicles produced in the endosome of eukaryotic cells, in the development, diagnosis, and treatment of AD has been recognized; however, their specific functions remain controversial and even unclear. With the development of exosome extraction, isolation, and characterization, many studies have focused on exosomes derived from different cells and body fluids. In this study, we summarized the roles of exosomes derived from different body fluids and cells, such as neuron, glial, stem, and endothelial cells, in the development, diagnosis, monitoring, and treatment of AD. We also emphasize the necessity to focus on exosomes from biological fluids and specific cells that are less invasive to target. Moreover, aside from the concentrations of classic and novel biomarkers in exosomes, the size and number of exosomes may also influence early and differential diagnosis of AD.

## Introduction

Alzheimer's disease (AD) is one of the leading causes of death worldwide and the most common form of dementia, comprising 60–80% of all cases (Landeiro et al., 2018). The global number of patients with AD is expected to rise to 65.7 million in 2030 and 115.4 million in 2050, at a rate of one new case every 3 s (Prince et al., 2013). AD is characterized by the accumulation of amyloid plaques formed by extracellular aggregates of the amyloid-β (Aβ) peptide, neurofibrillary tangles formed of intracellular hyperphosphorylated microtubule-associated tau proteins, and axonal degeneration. It can affect memory, use of language, and behavior and may develop into a severe disability, causing a huge burden to both families and society (Ghidoni et al., 2018). Currently, more than 99% of clinical trials for AD therapy have failed (Cummings et al., 2014), and only seven FDA-approved palliative drugs are available. Therefore, timely diagnosis and intervention are important in the early stages of AD.

According to the 2011 guidelines from the National Institute on Aging and the Alzheimer's Association (Jack et al., 2011), reliable biomarkers for AD only included the levels of Aβ1-42, total tau, and phosphorylated tau in the cerebrospinal fluid (CSF), which are expensive, invasive, and infeasible for screening. It has been estimated that, even in high-income countries, only 20–50% of dementia cases were correctly recognized and documented in primary care (Zheng et al., 2016). However, neuropathological alterations in patients with AD were reported to begin 10–20 years before the development of clinical symptoms (Villemagne et al., 2013). Thus, it is necessary and meaningful to find reliable and feasible biomarkers to help effectively diagnose AD, especially in the early stages.

In the last decade, many studies have found that extracellular vehicles (EVs) play key roles in the management of normal physiological environments, including waste management, stem cell maintenance, and tissue repair, as well as in pathological processes, including AD (Ratajczak et al., 2006; Gatti et al., 2011; van der Pol et al., 2012; Watson et al., 2019). Exosomes, 30–150 nm in diameter, are main EVs that originate in the endosome, and can carry multifarious molecular cargo, such as nucleic acids, lipids, and proteins (DeLeo and Ikezu, 2018; Yokoi et al., 2019). In the central nervous system, exosomes are critical for intercellular communication, maintenance of myelination, synaptic plasticity, and trophic support of neurons (Hornung et al., 2020). Currently, exosomes play important roles in the development, diagnosis, and treatment of AD; however, their specific functions remain controversial and even unclear. For example, some study found that exosomes could help spread tau proteins and encourage Aβ aggregation (Song et al., 2020a; Ruan et al., 2021); however, other studies reported that neuronal exosomes could restrain Aβ oligomerization, accelerate Aβ fibril formation, and facilitate microglia-mediated Aβ clearance, implying that exosomes from different cell types might exhibit different effects on the development of AD (Yuyama et al., 2012, 2014; Asai et al., 2015; Dinkins et al., 2017). Since exosomes secreted from different cell types contain particular and different types of markers, it is possible to identify their origins and isolate them from a specific cell subgroup, and corresponding extraction and purification methods have also been developed in recent years (Goetzl et al., 2019; Hornung et al., 2020). Thus, it is feasible and meaningful to recognize, extract, and analyze specific cell-derived exosomes based on the features of the targeted diseases. Noticeably, because exosomes are the main type of EVs, many authors interchangeably used the terms EVs and exosomes (Hornung et al., 2020). Thus, we also include some important studies taking about EVs in this review. Moreover, it is still challenging for effective and specific extraction, isolation, and characterization of exosomes, thus, the mentioned exosomes in many cases could also contain small amounts of other EVs.

Considering the above and based on previous published studies, in this review we mainly discuss and summarize the roles of exosomes derived from different body fluids and cells in the development, diagnosis, monitoring, and treatment of AD, as well as the remaining challenges in this field.

## Exosomes from various body fluids provide possibilities for the early diagnosis and intervention of Alzheimer's disease

### Cerebrospinal fluid-derived exosomes

A review reported that a large percentage of modulated proteins originate from exosomes, most of which are involved in the growth, development, maturation, and migration of neurons and neurotransmitter-mediated cellular communication (Bastos et al., 2017). Thus, CSF-derived exosomes have been widely studied, and their proteome has been recognized as a potential new reservoir for biomarker discovery in neurological disorders, including AD (Street et al., 2012). It has also been reported that more than 400 unique proteins mainly involved in AD, as well as the aging and telomere length pathway, were considerably enriched in CSF-derived EVs (Muraoka et al., 2019), and some proteins such as HSPA1A, NPEPPS, and PTGFRN could be used to monitor the progression of mild cognitive impairment (MCI) converted to AD (Muraoka et al., 2020). Moreover, the levels of total tau and p-181-tau in CSF-derived EVs were not only positively correlated with, but also higher than those in total CSF in individuals with AD (Guix et al., 2018; Muraoka et al., 2019). Thus, CSF-derived exosomes (or EVs) play important roles in the early and differential diagnosis of AD. However, the techniques used to derive exosomes from CSF still require refinement to reduce volume and variability.

### Blood-derived exosomes

Since the collection of exosomes from the CSF is invasive, it is necessary and urgent to find less or non-invasive biomarkers from other body fluids. Exosomes consist of a lipid bilayer encapsulating the cytosol and have an efficient capability to cross the blood-brain barrier without the loss of their biomarkers, thereby reaching many biological fluids, such as blood, urine, saliva, and synovial fluid (Colombo et al., 2012). Thus, the exosomes in these body fluids could dynamically reflect the pathological changes occurring in some inaccessible sites, such as the brain, making them promising biomarkers for the first step of multistage diagnoses.

Of these different body fluids, plasma and serum are the most widely used for the extraction of exosomes. Interestingly, previous studies have shown that the levels of exosome-bound Aβ could correlate better with PET imaging of brain amyloid plaques and differentiate various clinical stages of dementia compared to unbound or total circulating Aβ (Lim et al., 2019). Further extensive analyses of abnormal protein levels in neural cell-derived exosomes could identify patients with AD up to a decade ahead of clinically detectable cognitive losses (Goetzl, 2020). These reports imply that the levels of biomarkers in brain-derived, blood-borne exosomes can better reflect alterations occurring in the brain (Guix et al., 2018; Lim et al., 2019). It was also reported that the levels of Aβ1-42, total tau, p-181-tau, and p-S396-tau in neuron-derived exosomes (NDEs) from plasma were highly correlated with those in CSF and could differentiate patients with AD from those with MCI and/or controls with an accuracy of up to 96.4% (Fiandaca et al., 2015; Jia et al., 2019). Moreover, it was reported that patients who eventually developed AD had considerably higher levels of 181-tau and/or p-S396-tau in their NDEs compared to their plasma, even 10 years prior to diagnosis, compared to those who did not develop AD (Fiandaca et al., 2015; Winston et al., 2016).

Apart from the classic biomarkers, many other promising biomarkers such as lysosomal proteins, GAP43, neurogranin, SNAP25, and synaptotagmin 1 in NDEs from plasma could predict the development of AD at least 5 years before cognitive impairment and differentiate AD from frontotemporal dementia with 95.8% accuracy (Goetzl et al., 2015a; Jia et al., 2021). Another study also found that neuro-protective transcription factors such as repressor element 1 silencing transcription factor, low-density lipoprotein receptor-related protein 6, and heat shock factor 1 in NDEs from plasma decreased 2–10 years before the onset of clinical AD symptoms, implying a possible early pathogenic contribution of increased neuronal susceptibility to neurotoxic proteins in AD rather than higher levels of pathogenic proteins (Goetzl et al., 2015b). Furthermore, miRNAs such as miR-9-5p, miR-598, miR-125b, miR-29, miR-342-3p, and miR-193b, which are highly stable and resistant to degradation in exosomes, could be promising biomarkers for early clinical diagnosis and monitoring of AD (Riancho et al., 2017; Chen et al., 2019; Dong et al., 2020). Recently, a meta-analysis found that exosome-derived markers in serum had a higher diagnostic value for AD and MCI than those in plasma, implying that isolating exosomes from serum is a more accurate and non-invasive detection method (Xing et al., 2021).

### Exosomes derived from urine and other body fluids

A pilot study found that the levels of Aβ1-42 and p-S396-tau in urinary exosomes were higher in patients with AD than in controls, suggesting that it is a promising non-invasive biomarker (Sun et al., 2019). Comprehensive proteomic profiling analysis identified 336 differentially expressed proteins, including 44 brain cell biomarkers in urinary exosomes, of which 22 were further verified. Notably, annexin 2 and clusterin were markedly decreased in AD mouse models compared to control mice (Song et al., 2020b). Moreover, 48 differentially expressed miRNAs in urinary exosomes, including 18 upregulated and 30 downregulated ones, were identified and verified prior to the identification of Aβ plaque deposition, which was predicted to display gene targets and important signaling pathways closely associated with AD pathogenesis (Song et al., 2021). Furthermore, other non-invasive body fluids, such as saliva-derived exosomes (Han et al., 2018; Rani et al., 2021) are also promising for the early diagnosis of AD. However, associated studies are limited and need to be further explored, and the collections of saliva samples needs to be further standardized in clinical application.

## Exosomes derived from different cells play different roles in the development, diagnosis, and treatment of Alzheimer's disease

**Figure 1:**
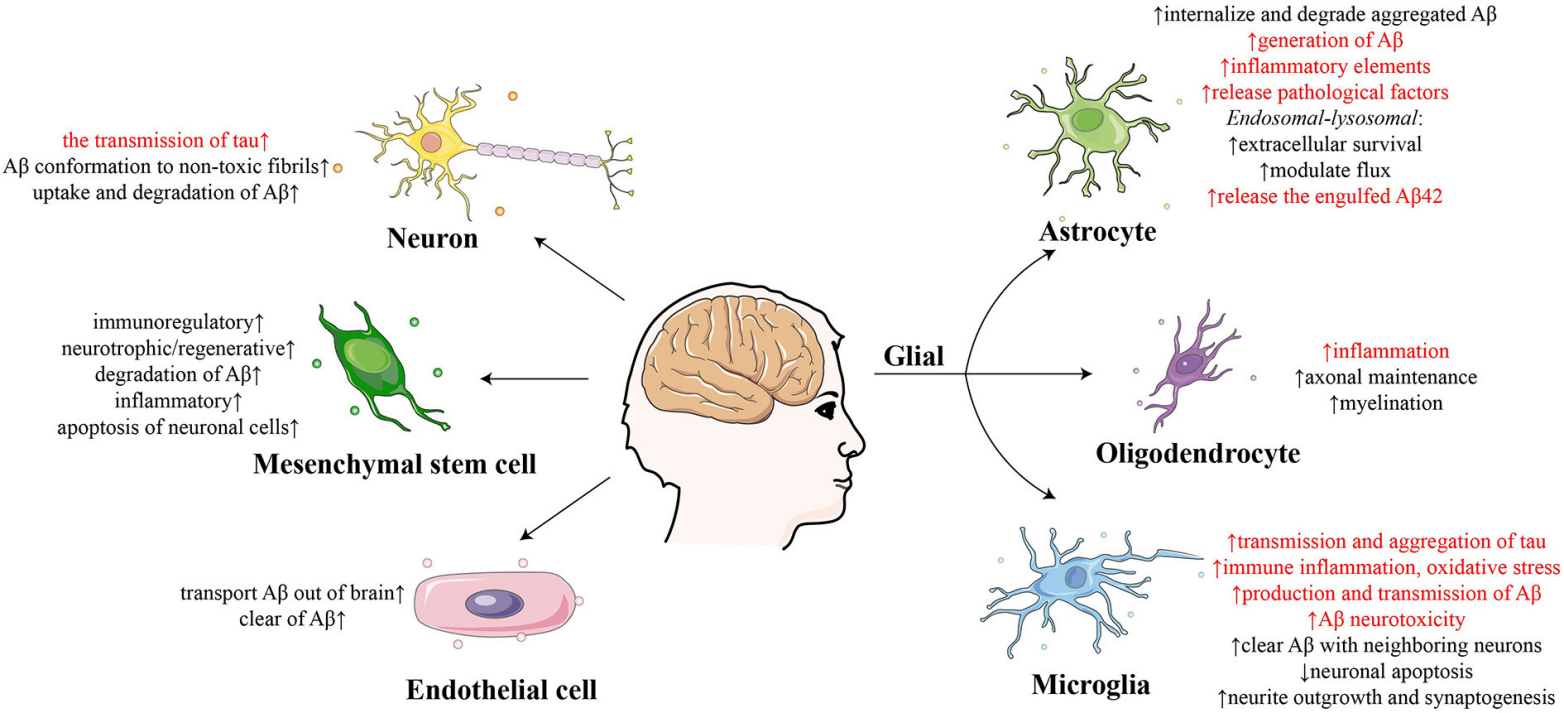
Summary of the effect of exosomes derived from different cells on the development of Alzheimer's disease.

Exosomes derived from neural cells play multifaceted roles in the nervous system, including in synaptic plasticity, the neuron-glia interface, neuroregeneration, neuroprotection, and the dissemination of neuropathological molecules (Upadhya et al., 2020). It has also been reported that exosomes derived from various types of neural cells, including neurons, glia, stem cells and so on, play different roles in the development of AD (Figure 1) (Song et al., 2020a); therefore, it is vital to distinguish and analyze specific cell-derived exosomes.

### Neuron-derived exosomes

As the basic structural and functional unit of the nervous system, neurons mediate various neural activities such as the conduction of excitation. Proteins in exosomes are released from affected neurons and propagate along neuroanatomically connected regions of the brain, leading to the spread of neurodegenerative diseases. Full-length tau proteins, which are more prone to aggregation, were found to be higher inside NDEs than in their free form (Guix et al., 2018), implying that NDEs could promote the transmission of tau. Moreover, the levels of P-S396-tau and P-T181-tau in NDEs can predict the development of AD up to 10 years before the clinical onset of sporadic AD (Fiandaca et al., 2015). Furthermore, a recent study demonstrated that NDEs, but not total exosomes, in plasma samples of AD patients could induce complement-mediated neurotoxicity, leading to the decrease of cell viability (Nogueras-Ortiz et al., 2020).

However, beneficial effects of NDEs on extracellular Aβ have also been found. NDEs may drive conformational changes in Aβ to form non-toxic amyloid fibrils and promote uptake and degradation by microglia (Yuyama et al., 2012). With enriched expression of glycosphingolipids, ceramide, and the GPI-anchored protein PrPc, NDEs showed stronger affinity for Aβ than other origin-derived exosomes, such as those from glial cells (Joshi et al., 2015; Yuyama et al., 2015). Moreover, it was reported that intracerebral infusion of NDEs into mouse brains could decrease the levels of Aβ and attenuate Aβ-induced synaptic density toxicity in the hippocampus (Yuyama et al., 2014, 2015), which implies that supplementation or promotion of NDE generation could be a novel therapeutic approach for AD (Yuyama et al., 2019).

Moreover, NDEs can also be used to diagnose AD. A meta-analysis showed that the area under the curve (AUC, 95% confidence interval) of miRNA and other biomarkers in NDEs for the diagnosis of AD or MCI was up to 0.89 (0.86–0.92), and a sub-group analysis found that NDEs from plasma had a higher AUC value (Xing et al., 2021). Higher levels of alpha-globin, beta-globin, and delta-globin in NDEs were found in patients with AD compared to those in controls (Arioz et al., 2021). The levels of presynaptic proteins, including synaptotagmin and synaptophysin, as well as postsynaptic proteins, including synaptopodin and neurogranin, in plasma NDEs were reported to be markedly lower in patients with AD, which was also correlated with the extent of cognitive loss (Goetzl et al., 2016a, 2018a). Many other biomarkers in NDEs were also found to be markedly different between patients with AD and controls, such as the level of Ser/Tyr phosphorylation of the insulin receptor substrate 1 (indicating insulin resistance), lysosomal enzymes and ubiquitin (indicating lysosomal dysfunction), and cellular survival factors (indicating impaired cellular stress responses) (Goetzl et al., 2015a,b; Kapogiannis et al., 2015). Furthermore, with the specific expression of neuronal, L1, or neural cell adhesion molecules, NDEs could be effectively isolated from total plasma/serum exosomes using a precipitation or immunoaffinity method with according antibodies (Fiandaca et al., 2015). With this isolation method, NDEs were found to comprise up to 15% of total plasma exosomes (Kapogiannis et al., 2015).

### Glial cell-derived exosomes

Brain function depends on coordinated interactions between neurons and glial cells, including astrocytes, microglia, and oligodendrocytes. However, the specific functions of glial cells and their derived exosomes remain controversial. For example, it has been reported that exosomes in glia could be transferred into neurons via cargo molecule-dependent and membrane contact-dependent mechanisms, which could benefit neurons or reversely disseminate the disease (Brites and Fernandes, 2015).

#### Astrocyte-derived exosomes

As the most abundant type of glial cells in the brain, astrocytes accumulate at sites of Aβ peptide deposition, internalizing and degrading aggregated peptides, which is considered a protective process (Wyss-Coray et al., 2003). Cellular experiments have shown that Aβ1-42 can inhibit exosome release from astrocytes by activating the c-Jun N-terminal kinase signaling pathway, thereby increasing the levels of astrocyte-derived exosomes (ADEs) via ultrasound stimulation can reverse oligomeric Aβ-induced cytotoxicity and help the clearance of Aβ plaques *in vitro* (Abdullah et al., 2016; Deng et al., 2021). Moreover, neuroprotective proteins such as synapsin 1, angiogenesis-associated molecules such as vascular endothelial growth factor, and matrix metalloproteinases involved in extracellular matrix proteolysis have been found in ADEs or astrocyte-derived EVs (Proia et al., 2008; Sbai et al., 2010; Wang et al., 2011). ADEs could also prolong the extracellular survival of protective endosomal-lysosomal cargo, such as the cysteine-protease inhibitor cystatin C, with their limiting membrane (Mathews and Levy, 2019). Efficient exosome release would help modulate flux via the neuronal–endosomal pathway by decompressing potential “traffic jams” and contributing to the degradation of neuronal debris (Mathews and Levy, 2019).

However, when a large amount of Aβ accumulates within astrocytes for a prolonged period without degradation, severe endosomal–lysosomal defects would occur in the astrocytes. Astrocytes can then release engulfed Aβ1-42 protofibrils through exosomes, leading to severe neurotoxicity to neighboring neurons (Söllvander et al., 2016). Animal experiments have also revealed that Aβ and tau released into the serum are most likely from ADEs in the brain (Rosas-Hernandez et al., 2019). Moreover, reducing the secretion of ADEs by neutral sphingomyelinase 2 loss-of-function could improve pathology and cognition in the 5XFAD mouse model (Dinkins et al., 2016). Recently, it was reported that some subsets of astrocytes contain amyloid precursor proteins (APP), β-secretase, and γ-secretase, which are required for the generation of Aβ peptides. These components are increased by fibrillary Aβ1-42 and several inflammatory cytokines (Zhao et al., 2011; Goetzl et al., 2016b). Furthermore, certain inflammatory and neurodegenerative reactions would elicit a coordinated response, leading to astrocyte hyperplasia and their conversion into reactive phenotypes, which could increase the expression of pro-inflammatory elements and damage both synapses and neurons (Liddelow and Barres, 2017; Goetzl et al., 2018b). Astrocytes can also release pathological factors such as S100β, a protein enriched in the brain of patients with AD that contributes to peri-plaque pathology (Mrak et al., 1996). Recently, a deep RNA sequencing study reported that ADEs influence neurodegenerative diseases mainly through metabolic and ubiquitin-dependent protein balance (Xie et al., 2022). Therefore, it is hard to deem the effect of astrocytes and ADEs on the development of AD as either “protective” or “disruptive.”

Currently, studies on the ability of classic biomarkers in ADEs to help in the diagnosis of AD are still lacking. The levels of complement proteins such as C1q, C3b, C3d, and cytokines including IL-6, TNF-α, and IL-1β in ADEs were found to be considerably different between patients with AD and controls, which could also distinguish between moderate and preclinical stage AD (Goetzl et al., 2018b; Goetzl, 2020). As components of neurotoxic neuroinflammation, complement proteins in plasma ADEs could be predictive biomarkers of patients with MCI progressing into AD, with considerably higher levels of C1q, C4b, factor D, fragment Bb, C5b, C3b, and C5b-C9 in converters within 3 years (Winston et al., 2019). The expression of the anti-human glutamine aspartate transporter has been widely used for the isolation of ADEs (Goetzl et al., 2018b). Moreover, plasma ADEs prominently express higher levels of glial fibrillary acidic protein and glutamine synthetase than NDEs, which could be used to distinguish between ADEs and NDEs (Goetzl et al., 2016b). Since, the cargos in exosomes derived from human neuron and astrocyte cells were mainly recognized as useful biomarkers for the diagnosis of AD, thus related results from previous studies were summarized in Table 1.

**Table 1:** Exosomes derived from human neuron and astrocyte cells as AD biomarkers in previous studies.

	**References**	**Disease group**	**Control group**	**Body fluid**	**Cell source**	**Isolation method**	**Validation techniques**	**Cargo change**
1	Fiandaca et al. (2015)	AD, *n =* 57	Cognitively normal controls, *n =* 57	Serum	Neuron	anti-NCAM	ELISA	↑total tau, P-S396-tau, P-T181-tau, Aβ1-42
2	Goetzl et al. (2016a)	AD, *n =* 24	Cognitively normal (1–10 years before the diagnosis of AD), *n =* 24	EDTA plasma	Neuron	anti-L1CAM	ELISA	↑P-S396-tau, P-T181-tau, Aβ1-42
3	Winston et al. (2016)	AD, *n =* 10	Cognitively normal controls, *n =* 10	Plasma	Neuron	anti-L1CAM	ELISA	↑P-S396-tau, P-T181-tau, Aβ1-42 ↓neurogranin, repressor element 1-silencing transcription factor
		Progressed to AD within 36 months, *n =* 20	Stable MCI, *n =* 20					
4	Jia et al. (2019)	AD, *n =* 28	Amnestic MCI, *n =* 15; healthy controls, *n =* 29	Plasma	Neuron	anti-NCAM	ELISA	↑Aβ42, T-tau, P-T181-tau
5	Arioz et al. (2021)	AD, *n =* 20	Healthy controls, *n =* 20	Serum	Neuron	anti-L1CAM	LC-MS/MS	↑alpha-globin, beta-globin, delta-globin
6	Goetzl et al. (2017)	AD, *n =* 28	Cognitively normal controls, *n =* 28	EDTA plasma	Neuron	anti-L1CAM	ELISA	↓neuronal pentraxin 2, neurexin 2a, GluA4-containing glutamate receptor, neuroligin 1
		AD, *n =* 18	Cognitively normal (6–11 years before the diagnosis of AD), *n =* 18					neurexin 2a, GluA4-containing glutamate receptor, neuroligin 1
7	Goetzl et al. (2016b)	AD, *n =* 12	Cognitively normal controls, *n =* 28	EDTA plasma	Neuron	anti-L1CAM	ELISA	↓synaptophysin, synaptopodin, synaptotagmin, neurogranin, growth-associated protein 43
		AD, *n =* 9	Cognitively intact or with MCI (1–10 years before the diagnosis of AD), *n =* 9					
8	Kapogiannis et al. (2015)	AD, *n =* 26	AD normal controls, *n =* 26	Plasma	Neuron	anti-L1CAM	ELISA	↑P-serine 312-IRS-1 ↓P-pan-tyrosine-IRS-1
		AD, *n =* 22	Cognitively normal (1–10 years before the diagnosis of AD), *n =* 22					
9	Goetzl et al. (2015a)	AD, *n =* 26	AD normal controls, *n =* 26	Plasma	Neuron	anti-L1CAM	ELISA	↑cathepsin D, lysosome-associated membrane protein 1, ubiquitinylated proteins ↓heat-shock protein 70
		AD, *n =* 20	Cognitively normal (1–10 years before the diagnosis of AD), *n =* 20					
10	Goetzl et al. (2015b)	AD, *n =* 24	AD normal controls, *n =* 24	Heparin plasma	Neuron	anti-L1CAM	ELISA	↓low-density lipoprotein receptor-related protein 6, heat-shock factor-1, repressor element 1-silencing transcription factor
		Preclinical AD (1–10 years before diagnosis of AD), *n =* 16	Cognitively normal controls, *n =* 16					
11	Goetzl et al. (2016a)	Amnestic MCI or early AD, *n =* 12	Cognitively normal controls, *n =* 10	EDTA plasma	Astrocyte	anti-ACSA-1	ELISA	*↑β*-secretase 1, sAPPb, Aβ42 ↓septin-8, GDNF
12	Goetzl et al. (2018a)	Early AD, *n =* 28	Cognitively normal controls, *n =* 28	EDTA plasma	Astrocyte	anti-ACSA-1	ELISA	↑IL-6, TNF-α, IL-1β, C1q, C4b, factor B, factor D, Bb, C3b, C3d, C5b-C9 terminal complement complex ↓CR1, CD46, CD59, DAF
		AD, *n =* 16	Cognitively normal (5–12 years before the diagnosis of AD), *n =* 16					↑C4b, C3d, factor B, Bb, C3b, C5b-C9 terminal complement complex, TNF-α, IL-1β ↓CD59, DAF
13	Winston et al. (2019)	AD, *n =* 20	Cognitively normal controls, *n =* 20	EDTA plasma	Astrocyte	anti-ACSA-1	ELISA	↑C1q, C4b, factor D, Bb, C5b, C3b, C5b-C9
		Progressed to AD within 3 years, *n =* 20	Stable MCI during the 36-month study, *n =* 20					

*AD, Alzheimer's disease; MCI, mild cognitive impairment; L1CAM, L1 cell adhesion molecule; NCAM, neural cell adhesion molecule; ACSA-1, human glutamine aspartate transporter*.

#### Microglia-derived exosomes

As the primary innate immune cells in the brain, microglia can detect tissue damage and microbial infection, and phagocytose not only dying cells and, protein aggregates, but living neurons, and synapses (Neumann et al., 2009; Schafer et al., 2012). It was also found that exosomes added to mixed brain cultures containing all major cell types were preferentially taken up by microglia (Fitzner et al., 2011). Moreover, microglial-derived EVs could influence neurite outgrowth and modulate neuronal activity (Delpech et al., 2019; Paolicelli et al., 2019). However, it is relatively difficult to define the function of microglia as “protective” or ‘disruptive”, because they can dynamically switch between different phenotypes depending on the stage of the disease (Guo et al., 2021).

Microglia can phagocytize tau-containing cytopathic neurons or synapses and efficiently transmit tau to neurons via phagocytosis and exosomes. Thus, microglia and microglia-derived exosomes (MDEs) are positively correlated with tau pathology, and their depletion dramatically suppresses the propagation of tau and reduces excitability in the dentate gyrus (Schafer et al., 2012; Asai et al., 2015; Ruan et al., 2020). When tau-containing MDEs are taken up by neurons, abnormal aggregation of tau is further triggered (Asai et al., 2015). Moreover, reactive microglia may release exosomes carrying the inflammatory markers, including iNOS, IL-1β, TNF-α, MHC class II, IL-6, miR-155, miR-146a and miR-124, and pro-resolving genes, including IL-10 and arginase 1, leading to a more damaging pro-inflammatory state throughout the brain (Frühbeis et al., 2013; Fernandes et al., 2018). Deep RNA sequencing also demonstrated that MDEs influence neurodegenerative diseases through immune inflammation and oxidative stress (Xie et al., 2022). Suppressing the expression of miR-21-5p is considered a promising novel strategy for the treatment of neuroinflammation (Yin et al., 2020). Furthermore, it was demonstrated that MDEs and microglia-derived EVs not only had higher APP and Aβ1-40 production, carried oligomeric Aβ, and mediated Aβ transmission, but also promoted the extracellular aggregation of Aβ1-42 to form small soluble neurotoxic species, which strongly increased Aβ neurotoxicity (Joshi et al., 2014; Fernandes et al., 2018; Gouwens et al., 2018). However, it has been reported that microglia and neighboring neurons could work together to clear Aβ peptides via exosomes (Guo et al., 2021). Microglia-depleted mice had increased levels of soluble Aβ in the brain, and statins could promote the degradation of extracellular Aβ by stimulating the secretion of MDE-associated insulin-degrading enzymes, indicating a beneficial role of microglia in the metabolism of extracellular Aβ (Fuhrmann et al., 2010; Tamboli et al., 2010). Furthermore, cargo molecules in microglia derived EVs, including trombospondin-1 and 4, can suppress neuronal apoptosis and promote neurite outgrowth and synaptogenesis, implying a neuroprotective role (Drago et al., 2017). Thus, further investigation of the roles of microglia and MDEs in central nervous system disorders is required.

Recently, advances in single-cell RNA sequencing have helped unravel some questions in the field of microglia through the discovery of a new phenotype called “disease-associated microglia.” These accumulate around plaques and exhibit upregulated gene expression of apolipoprotein E (APOE) and trigger the receptors expressed on myeloid cells 2 (TREM2), which are considered genetic risk factors for AD (Keren-Shaul et al., 2017). However, another study found that TREM2 knockout ameliorated amyloid pathology in the early stages of AD, but exacerbated it later in the disease process (Jay et al., 2017), which highlights the roles of microglia and MDEs in AD pathology depending on the stage of the disease. Furthermore, a reproducible and efficient method for yielding purified primary microglia cells and effectively isolating and characterization of MDEs based on CD11b/c has been proposed in recent years (Murgoci et al., 2018), which is beneficial for further studies on MEDs.

#### Oligodendrocyte-derived exosomes

Although few studies have focused on the potential roles of oligodendrocytes in AD pathogenesis, some gene variants that increase the risk of AD have been found to be predominantly expressed in oligodendrocytes. Interact with axon, oligodendrocyte played an important role in neuronal integrity. Oligodendrocyte-derived exosomes (ODEs) may be internalized by neurons through endocytosis, which could contribute to neuroprotection and long-term axonal maintenance under normal, or oxygen and glucose-deprived conditions (Frühbeis et al., 2012; Fröhlich et al., 2014). Moreover, it was reported that markedly increased numbers of both newly generated and mature oligodendrocytes following treatment with mesenchymal stromal cell-derived exosomes considerably decreased amyloid-β precursor protein density and improved neurological outcomes (Zhang et al., 2021). ODEs can also participate in the management of oxidative stress by transferring human superoxide dismutase and catalase. When stress resistance occurs following ischemia, neurons take up more ODEs (Krämer-Albers et al., 2007). Notably, alterations in the composition of ODEs under some pathological conditions may switch immunologically inert exosomes into active ones, which may trigger inflammatory reactions in the brain. Furthermore, an immunocapture protocol based on common oligodendrocyte biomarkers, such as 2,3-cyclic nucleotide-3-phosphodiesterase, can extract and isolate ODEs for further study (Yu et al., 2020).

### Stem cell-derived exosomes

Stem cell-derived exosomes play important roles in the therapy for AD (Vasic et al., 2019; Sivandzade and Cucullo, 2021). Compared with traditional stem cell transplantation, stem cell-derived exosomes are relatively easier to manage, have lower immunogenicity, and have a lower risk of tumor formation, making them a potential therapeutic method (Guo et al., 2020). When the unique functionalities of exosome-derived membranes are combined with synthetic gold nanoparticles (AuNPs), efficient brain targeting can be achieved (Khongkow et al., 2019). In 5xFAD accelerated transgenic mouse model of AD, human neural stem cell-derived EVs showed the regenerative potential on the neurocognitive and neuropathologic hallmarks, and significantly reduced dense core Aβ plaque accumulation and microglial activation in the AD brain (Apodaca et al., 2021).

Mesenchymal stem cells (MSCs), a type of adult pluripotent stem cell, are derived from connective tissue especially adipose tissue. MSCs can reduce the Aβ plaque burden by the internalization and degradation of Aβ oligomers via the endosomal-lysosomal pathway. Electron microscopy and proteomic analysis further revealed that the therapeutic effect of MSCs was due to their exosomes (Lai et al., 2010). With well-characterized immunoregulatory neurotrophic and regenerative properties, MSC-derived exosomes were considered as a promising candidate for AD therapy and most widely studied. In mouse models, MSC-derived exosome administration via the lateral ventricle or intravenous injection could reduce Aβ expression and improve AD-like behaviors (Chen et al., 2021; Liu et al., 2022). Enzymatically active neprilysin, the most important Aβ-degrading enzyme in the brain, is also found in MSC-derived exosomes (Joshi et al., 2015). Moreover, MSC-derived EVs were considered as the best cell-free candidates for promoting a reparative process by activating positive responses in the brain microenvironment via intercellular communication (Elia et al., 2019). MiRNA-22-loaded exosomes derived from adipose-derived MSCs can decrease the release of inflammatory factors, thereby playing a synergetic therapeutic role in AD (Zhai et al., 2021). Moreover, miR-223 loaded MSC-derived exosomes protected neuronal cells from apoptosis via the PTEN-PI3K/AKT pathway, providing a potential therapeutic approach for AD (Wei et al., 2020). Furthermore, a previous study proved the safety of MSC-derived EVs (Nassar et al., 2016), and a clinical trial was conducted to evaluate their safety and effectiveness in patients with mild to moderate dementia (www.clinicaltrials.gov, NCT04388982). It is worth noting that the efficacy was dosage-dependent, and the lower dose of exosomes was found to be more neuroprotective (Venugopal et al., 2017). Moreover, specific MSC-related molecules, such as CD29, CD44, CD90, and CD73, could help recognize and isolate MSC-derived exosomes.

Noticeably, even if EVs derived from native stem cells have potential in the treatment of neurodegenerative diseases including AD, its clinical application is still limited due to the short half-life, limited targeting, rapid clearance after application, and insufficient payload (Bang and Kim, 2022). Some strategies such as engineered EVs by genetic modification could improve stability, targeting ability and EVs tracking (Lino et al., 2021), thus, associated technologies need to be further explored and developed to prompt the clinical therapeutic application of stem cell-derived exosomes.

### Endothelial cell-derived exosomes

Exosomes derived from endothelial cells (EDEs) of the human brain microvasculature contain P-glycoprotein, a member of the ABC transporter family, which can effectively transport Aβ out of the brain. Thus, it has been reported that EDEs can greatly facilitate the cerebral clearance of Aβ and potently ameliorate cognitive dysfunction in AD mice (Pan et al., 2020). CD81-normalized levels of Aβ1-40 and Aβ1-42 in plasma EDEs were also found to be considerably higher in the preclinical AD/MCI group with small cerebral vascular disease than in controls, which often occurs before the presentation of neuronal and other cellular changes in AD (Abner et al., 2020). Moreover, endothelial proteins such as vascular cell adhesion molecule-1 and endothelial nitric oxide synthase can be used for the precipitation and enrichment of EDEs when using immune-specific absorption procedures for the analysis of cargo proteins (Goetzl et al., 2017).

## The size and number of exosomes could be promising biomarkers for the diagnosis of AD

A recent study found that not only the constitution Aβ and p-S396-tau in exosomes markedly differed between patients with AD compared to the controls, but the exosomes from patients with AD were smaller and lower in quantity, as determined by transmission electron microscopy (TEM) and nanoparticle tracking analysis (NTA), contributing to the early diagnosis of AD (Sun et al., 2020). Since TEM and NTA technology are widely used to visualize and characterize extracted exosomes, it is feasible to combine information on the size and number of exosomes with biomarker levels for the early and differential diagnosis of AD (Szatanek et al., 2017).

### The size of exosomes

One study reported that the size of plasma exosomes was smaller in an AD group than in the control group, which is beneficial for early diagnosis of AD (Sun et al., 2020). However, some studies found larger size of plasma EVs in patients with AD than in controls, which could be induced by the uptake and accumulation of Aβ (Longobardi et al., 2021). Moreover, in another study, no significant difference was observed in the diameters of EVs in the CSF between patients with AD and the controls (Saugstad et al., 2017). These inconsistent results are likely to be due to the differences in exosome types and sources. Thus, further studies need to be conducted to explore the roles of specific exosomes in the diagnosis of AD.

### The number of exosomes

Some studies have reported markedly lower concentrations of plasma exosomes in patients with AD compared to those in controls (Sun et al., 2020; Longobardi et al., 2021). Considering both the size and concentration of exosomes, the diagnostic performance to distinguish between dementia and controls was high, with a sensitivity of 83.3% and specificity of 86.7% (Longobardi et al., 2021). Moreover, APOE4 was found to drive the downregulation of brain exosome biosynthesis and release, which plays an important role in endosomal and lysosomal deficits and could lead to a higher risk of AD development (Peng et al., 2019). However, other studies found that astrocytes in AD could induce an increased release of exosomes containing toxic proteins, and exposure to amyloid *in vitro* could increase the production of cell-derived exosomes (Dinkins et al., 2014; Chiarini et al., 2017). Another study found that the number of serum exosomes in transgenic mice with AD was considerably higher than that in wild-type mice, with increased ADEs and decreased EDEs (Rosas-Hernandez et al., 2019). Moreover, NDEs from participants with Down syndrome, who had characteristic neuropathological features of AD at the age of 40 years and eventually developed AD, had 39% higher levels of exosomes on average than those from the control group [(1,433 ± 87 pg/ml) vs. (1,027 ± 87 pg/ml)] (Hamlett et al., 2018). It was also reported that upregulation of exosome release was recognized as a useful mechanism to help clear these deleterious proteins, which could also be recognized in urine samples (Sun et al., 2019). Furthermore, a basic study found that preventing exosome secretion could reduce the formation of amyloid plaques *in vivo*; thus, drugs interfering with exosome secretion, such as neutral nSMase2, could be used as potential drug targets in AD (Dinkins et al., 2014, 2015).

These inconsistent results could also be related to the differences in exosome types and sources, as well as different efficacies of extraction across different samples. Thus, more studies are needed to reach a definite conclusion in which the standardized process of exosome extraction, isolation, and characterization plays an important role. Moreover, the released exosomes were mostly recognized by the CD81 marker, which could be interfered with possible increased soluble CD81 levels per exosome. Thus, an accurate count of exosome numbers is important. NTA using ALIX as an exosome marker or other burgeoning high-sensitivity exosome-counting systems, such as Exo-counter and single molecule array, has been proven to be more accurate for the determination of exosome amounts (Fiandaca et al., 2015; Kapogiannis et al., 2015; Eitan et al., 2017; Yokose et al., 2020; Ter-Ovanesyan et al., 2021).

## Conclusion

The role of specific cell-derived exosomes in the development, diagnosis, monitoring, and treatment of AD has attracted increasing attention in recent years. However, the conclusions of different studies are largely inconsistent and many mechanisms remain unclear. Thus, we summarize the roles of exosomes derived from different body fluids and cells in the development, diagnosis, monitoring, and treatment of AD in this study, and emphasize the necessity to focus on exosomes from specific cells and less-invasive biological fluids. Moreover, aside from the concentrations of classic and novel biomarkers in exosomes, we recognized and summarized the roles of the size and number of exosomes play in early and differential diagnosis of AD at first.

Interestingly, a recent study pointed out that a few proteins that had not yet been reported to be expressed in neurons were highly expressed in NDEs, implying that some NDEs may originate in non-neuronal tissues (Pulliam et al., 2019). Thus, the process and mechanism of specific cell-derived exosomes, from their origin, formation, and transportation to their ultimate roles in AD, still need to be further studied. Second, the standardization and automation of the whole process of extraction, isolation, and characterization, including the size and number of specific cell-derived exosomes, remain a challenge (Doyle and Wang, 2019; Guo et al., 2020; Hornung et al., 2020) and need to be developed and optimized. Moreover, methods which is time-saving, low-cost, and convenient, must be explored for clinical applications. Furthermore, it is crucial to accurately measure the levels of promising biomarkers in exosomes, particularly classical Aβ and tau, which are of great importance for their clinical application. The levels of Aβ1-42 in NDEs are <10 pg/ml, and that of p-181-tau is ~100 pg/ml, which is difficult to accurately determine with the traditional ELISA method. Therefore, automatic detection platforms, such as electrochemiluminescence instruments and single molecule arrays, and technologies with stronger anti-interference abilities, such as mass spectra, need to be developed. Except for the exosomes in CSF, further studies should focus on specific cell-derived exosomes in plasma, urine, and other non-invasive fluids, considering not only their internal biomarkers, but also their sizes and numbers.

## Author contributions

LQ and SY mainly guided the study. YZ mainly wrote this manuscript. DM mainly drawn the figure. XM, JZ, DW, and JG made suggestions for revision of the manuscript. All authors reviewed the manuscript and approved the submission.

## Funding

This study was supported by National Key Research and Development Program of China (No. 2021YFC2009300/2021YFC2009302), National Key Research and Development Program of China (No. 2020YFA0804501), Education Reforming Funding of PUMC (10023201900101), and Beijing Key Clinical Specialty for Laboratory Medicine - Excellent Project (No. ZK201000).

## Conflict of interest

The authors declare that the research was conducted in the absence of any commercial or financial relationships that could be construed as a potential conflict of interest.

## Publisher's note

All claims expressed in this article are solely those of the authors and do not necessarily represent those of their affiliated organizations, or those of the publisher, the editors and the reviewers. Any product that may be evaluated in this article, or claim that may be made by its manufacturer, is not guaranteed or endorsed by the publisher.

## References

[B1] Abdullah M. Takase H. Nunome M. Enomoto H. Ito J. Gong J. S.. (2016). Amyloid-β reduces exosome release from astrocytes by enhancing JNK phosphorylation. J. Alzheimers Dis. 53, 1433–1441. 10.3233/JAD-160292 27392863

[B2] Abner E. L. Elahi F. M. Jicha G. A. Mustapic M. Al-Janabi O. Kramer J. H.. (2020). Endothelial-derived plasma exosome proteins in Alzheimer's disease angiopathy. FASEB J. 34, 5967–5974. 10.1096/fj.202000034R 32157747 PMC7233139

[B3] Apodaca L. A. Baddour A. A. D. Garcia C. Jr. Alikhani L. Giedzinski E. Ru N.. (2021). Human neural stem cell-derived extracellular vesicles mitigate hallmarks of Alzheimer's disease. Alzheimers Res. Ther. 13:57. 10.1186/s13195-021-00791-x 33676561 PMC7937214

[B4] Arioz B. I. Tufekci K. U. Olcum M. Durur D. Y. Akarlar B. A. Ozlu N.. (2021). Proteome profiling of neuron-derived exosomes in Alzheimer's disease reveals hemoglobin as a potential biomarker. Neurosci. Lett. 755:135914. 10.1016/j.neulet.2021.135914 33901610

[B5] Asai H. Ikezu S. Tsunoda S. Medalla M. Luebke J. Haydar T.. (2015). Depletion of microglia and inhibition of exosome synthesis halt tau propagation. Nat. Neurosci. 18, 1584–1593. 10.1038/nn.4132 26436904 PMC4694577

[B6] Bang O. Y. Kim J. E. (2022). Stem cell-derived extracellular vesicle therapy for acute brain insults and neurodegenerative diseases. BMB Rep. 55, 20–29. 10.5483/BMBRep.2022.55.1.162 35000673 PMC8810548

[B7] Bastos P. Ferreira R. Manadas B. Moreira P. I. Vitorino R. (2017). Insights into the human brain proteome: Disclosing the biological meaning of protein networks in cerebrospinal fluid. Crit. Rev. Clin. Lab. Sci. 54, 185–204. 10.1080/10408363.2017.1299682 28393582

[B8] Brites D. Fernandes A. (2015). Neuroinflammation and depression: microglia activation, extracellular microvesicles and microRNA dysregulation. Front. Cell. Neurosci. 9:476. 10.3389/fncel.2015.00476 26733805 PMC4681811

[B9] Chen J. J. Yang G. Yan Q. Q. Zhao J. Li S. (2019). Exosome-encapsulated microRNAs as promising biomarkers for Alzheimer's disease. Rev. Neurosci. 31, 77–87. 10.1515/revneuro-2019-0001 31318699

[B10] Chen Y. A. Lu C. H. Ke C. C. Chiu S. J. Jeng F. S. Chang C. W.. (2021). Mesenchymal stem cell-derived exosomes ameliorate Alzheimer's disease pathology and improve cognitive deficits. Biomedicines 9:594. 10.3390/biomedicines9060594 34073900 PMC8225157

[B11] Chiarini A. Armato U. Gardenal E. Gui L. Dal Prà I. (2017). Amyloid β-exposed human astrocytes overproduce Phospho-Tau and overrelease it within exosomes, effects suppressed by calcilytic NPS 2143-further implications for Alzheimer's therapy. Front. Neurosci. 11:217. 10.3389/fnins.2017.00217 28473749 PMC5397492

[B12] Colombo E. Borgiani B. Verderio C. Furlan R. (2012). Microvesicles: novel biomarkers for neurological disorders. Front. Physiol. 3:63. 10.3389/fphys.2012.00063 22479250 PMC3315111

[B13] Cummings J. L. Morstorf T. Zhong K. (2014). Alzheimer's disease drug-development pipeline: few candidates, frequent failures. Alzheimers Res. Ther. 6:37. 10.1186/alzrt269 25024750 PMC4095696

[B14] DeLeo A. M. Ikezu T. (2018). Extracellular vesicle biology in Alzheimer's disease and related tauopathy. J. Neuroimmune Pharmacol. 13, 292–308. 10.1007/s11481-017-9768-z 29185187 PMC5972041

[B15] Delpech J. C. Herron S. Botros M. B. Ikezu T. (2019). Neuroimmune crosstalk through extracellular vesicles in health and disease. Trends Neurosci. 42, 361–372. 10.1016/j.tins.2019.02.007 30926143 PMC6486849

[B16] Deng Z. Wang J. Xiao Y. Li F. Niu L. Liu X.. (2021). Ultrasound-mediated augmented exosome release from astrocytes alleviates amyloid-β-induced neurotoxicity. Theranostics 11, 4351–4362. 10.7150/thno.52436 33754065 PMC7977450

[B17] Dinkins M. B. Dasgupta S. Wang G. Zhu G. Bieberich E. (2014). Exosome reduction in vivo is associated with lower amyloid plaque load in the 5XFAD mouse model of Alzheimer's disease. Neurobiol. Aging 35, 1792–1800. 10.1016/j.neurobiolaging.2014.02.012 24650793 PMC4035236

[B18] Dinkins M. B. Dasgupta S. Wang G. Zhu G. He Q. Kong J. N.. (2015). The 5XFAD mouse model of Alzheimer's disease exhibits an age-dependent increase in anti-ceramide IgG and exogenous administration of ceramide further increases anti-ceramide titers and amyloid plaque burden. J. Alzheimers Dis. 46, 55–61. 10.3233/JAD-150088 25720409 PMC4593501

[B19] Dinkins M. B. Enasko J. Hernandez C. Wang G. Kong J. Helwa I.. (2016). Neutral Sphingomyelinase-2 deficiency ameliorates Alzheimer's disease pathology and improves cognition in the 5XFAD mouse. J. Neurosci. 36, 8653–8667. 10.1523/JNEUROSCI.1429-16.2016 27535912 PMC4987436

[B20] Dinkins M. B. Wang G. Bieberich E. (2017). Sphingolipid-enriched extracellular vesicles and Alzheimer's disease: a decade of research. J. Alzheimers Dis. 60, 757–768. 10.3233/JAD-160567 27662306 PMC5360538

[B21] Dong X. Zheng D. Nao J. (2020). Circulating Exosome microRNAs as diagnostic biomarkers of dementia. Front. Aging Neurosci. 12:580199. 10.3389/fnagi.2020.580199 33093831 PMC7506134

[B22] Doyle L. M. Wang M. Z. (2019). Overview of extracellular vesicles, their origin, composition, purpose, and methods for exosome isolation and analysis. Cells 8:727. 10.3390/cells8070727 31311206 PMC6678302

[B23] Drago F. Lombardi M. Prada I. Gabrielli M. Joshi P. Cojoc D.. (2017). ATP modifies the proteome of extracellular vesicles released by microglia and influences their action on astrocytes. Front. Pharmacol. 8:910. 10.3389/fphar.2017.00910 29321741 PMC5733563

[B24] Eitan E. Tosti V. Suire C. N. Cava E. Berkowitz S. Bertozzi B.. (2017). In a randomized trial in prostate cancer patients, dietary protein restriction modifies markers of leptin and insulin signaling in plasma extracellular vesicles. Aging Cell 16, 1430–1433. 10.1111/acel.12657 28921841 PMC5676054

[B25] Elia C. A. Losurdo M. Malosio M. L. Coco S. (2019). Extracellular vesicles from mesenchymal stem cells exert pleiotropic effects on Amyloid-β, inflammation, and regeneration: a spark of hope for Alzheimer's disease from tiny structures? Bioessays 41:e1800199. 10.1002/bies.201800199 30919493

[B26] Fernandes A. Ribeiro A. R. Monteiro M. Garcia G. Vaz A. R. Brites D. (2018). Secretome from SH-SY5Y APP(Swe) cells trigger time-dependent CHME3 microglia activation phenotypes, ultimately leading to miR-21 exosome shuttling. Biochimie 155, 67–82. 10.1016/j.biochi.2018.05.015 29857185

[B27] Fiandaca M. S. Kapogiannis D. Mapstone M. Boxer A. Eitan E. Schwartz J. B.. (2015). Identification of preclinical Alzheimer's disease by a profile of pathogenic proteins in neurally derived blood exosomes: a case-control study. Alzheimers Dement. 11, 600–607.e1. 10.1016/j.jalz.2014.06.008 25130657 PMC4329112

[B28] Fitzner D. Schnaars M. van Rossum D Krishnamoorthy G Dibaj P Bakhti M (2011). Selective transfer of exosomes from oligodendrocytes to microglia by macropinocytosis. J. Cell Sci. 124, 447–458. 10.1242/jcs.074088 21242314

[B29] Fröhlich D. Kuo W. P. Frühbeis C. Sun J. J. Zehendner C. M. Luhmann H. J.. (2014). Multifaceted effects of oligodendroglial exosomes on neurons: impact on neuronal firing rate, signal transduction and gene regulation. Philos. Trans. R. Soc. Lond. B. Biol. Sci. 369:20130510. 10.1098/rstb.2013.0510 25135971 PMC4142031

[B30] Frühbeis C. Fröhlich D. Krämer-Albers E. M. (2012). Emerging roles of exosomes in neuron-glia communication. Front. Physiol. 3:119. 10.3389/fphys.2012.00119 22557979 PMC3339323

[B31] Frühbeis C. Fröhlich D. Kuo W. P. Amphornrat J. Thilemann S. Saab A. S.. (2013). Neurotransmitter-triggered transfer of exosomes mediates oligodendrocyte-neuron communication. PLoS Biol. 11:e1001604. 10.1371/journal.pbio.1001604 23874151 PMC3706306

[B32] Fuhrmann M. Bittner T. Jung C. K. Burgold S. Page R. M. Mitteregger G.. (2010). Microglial Cx3cr1 knockout prevents neuron loss in a mouse model of Alzheimer's disease. Nat. Neurosci. 13, 411–413. 10.1038/nn.2511 20305648 PMC4072212

[B33] Gatti S. Bruno S. Deregibus M. C. Sordi A. Cantaluppi V. Tetta C.. (2011). Microvesicles derived from human adult mesenchymal stem cells protect against ischaemia-reperfusion-induced acute and chronic kidney injury. Nephrol. Dial. Transplant 26, 1474–1483. 10.1093/ndt/gfr015 21324974

[B34] Ghidoni R. Squitti R. Siotto M. Benussi L. (2018). Innovative biomarkers for Alzheimer's disease: focus on the hidden disease biomarkers. J. Alzheimers Dis. 62, 1507–1518. 10.3233/JAD-170953 29504534

[B35] Goetzl E. J. (2020). Advancing medicine for Alzheimer's disease: a plasma neural exosome platform. FASEB J. 34, 13079–13084. 10.1096/fj.202001655 32856798

[B36] Goetzl E. J. Abner E. L. Jicha G. A. Kapogiannis D. Schwartz J. B. (2018a). Declining levels of functionally specialized synaptic proteins in plasma neuronal exosomes with progression of Alzheimer's disease. FASEB J. 32, 888–893. 10.1096/fj.201700731R 29025866 PMC5888398

[B37] Goetzl E. J. Boxer A. Schwartz J. B. Abner E. L. Petersen R. C. Miller B. L.. (2015a). Altered lysosomal proteins in neural-derived plasma exosomes in preclinical Alzheimer disease. Neurology 85, 40–47. 10.1212/WNL.0000000000001702 26062630 PMC4501943

[B38] Goetzl E. J. Boxer A. Schwartz J. B. Abner E. L. Petersen R. C. Miller B. L.. (2015b). Low neural exosomal levels of cellular survival factors in Alzheimer's disease. Ann. Clin. Transl. Neurol. 2, 769–773. 10.1002/acn3.211 26273689 PMC4531059

[B39] Goetzl E. J. Kapogiannis D. Schwartz J. B. Lobach I. V. Goetzl L. Abner E. L.. (2016a). Decreased synaptic proteins in neuronal exosomes of frontotemporal dementia and Alzheimer's disease. FASEB J. 30, 4141–4148. 10.1096/fj.201600816R 27601437 PMC5102122

[B40] Goetzl E. J. Mustapic M. Kapogiannis D. Eitan E. Lobach I. V. Goetzl L.. (2016b). Cargo proteins of plasma astrocyte-derived exosomes in Alzheimer's disease. FASEB J. 30, 3853–3859. 10.1096/fj.201600756R 27511944 PMC5067254

[B41] Goetzl E. J. Nogueras-Ortiz C. Mustapic M. Mullins R. J. Abner E. L. Schwartz J. B.. (2019). Deficient neurotrophic factors of CSPG4-type neural cell exosomes in Alzheimer disease. FASEB J. 33, 231–238. 10.1096/fj.201801001 29924942 PMC6355088

[B42] Goetzl E. J. Schwartz J. B. Abner E. L. Jicha G. A. Kapogiannis D. (2018b). High complement levels in astrocyte-derived exosomes of Alzheimer disease. Ann. Neurol. 83, 544–552. 10.1002/ana.25172 29406582 PMC5867263

[B43] Goetzl E. J. Schwartz J. B. Mustapic M. Lobach I. V. Daneman R. Abner E. L.. (2017). Altered cargo proteins of human plasma endothelial cell-derived exosomes in atherosclerotic cerebrovascular disease. FASEB J. 31, 3689–3694. 10.1096/fj.201700149 28476896 PMC5503715

[B44] Gouwens L. K. Ismail M. S. Rogers V. A. Zeller N. T. Garrad E. C. Amtashar F. S.. (2018). Aβ42 protofibrils interact with and are trafficked through microglial-derived microvesicles. ACS Chem. Neurosci. 9, 1416–1425. 10.1021/acschemneuro.8b00029 29543435

[B45] Guix F. X. Corbett G. T. Cha D. J. Mustapic M. Liu W. Mengel D.. (2018). Detection of aggregation-competent Tau in neuron-derived extracellular vesicles. Int. J. Mol. Sci. 19:663. 10.3390/ijms19030663 29495441 PMC5877524

[B46] Guo M. Hao Y. Feng Y. Li H. Mao Y. Dong Q.. (2021). Microglial exosomes in neurodegenerative disease. Front. Mol. Neurosci. 14:630808. 10.3389/fnmol.2021.630808 34045943 PMC8148341

[B47] Guo M. Yin Z. Chen F. Lei P. (2020). Mesenchymal stem cell-derived exosome: a promising alternative in the therapy of Alzheimer's disease. Alzheimers Res. Ther. 12:109. 10.1186/s13195-020-00670-x 32928293 PMC7488700

[B48] Hamlett E. D. Ledreux A. Potter H. Chial H. J. Patterson D. Espinosa J. M.. (2018). Exosomal biomarkers in Down syndrome and Alzheimer's disease. Free Radic. Biol. Med. 114, 110–121. 10.1016/j.freeradbiomed.2017.08.028 28882786 PMC6135098

[B49] Han Y. Jia L. Zheng Y. Li W. (2018). Salivary exosomes: emerging roles in systemic disease. Int. J. Biol. Sci. 14, 633–643. 10.7150/ijbs.25018 29904278 PMC6001649

[B50] Hornung S. Dutta S. Bitan G. (2020). CNS-derived blood exosomes as a promising source of biomarkers: opportunities and challenges. Front. Mol. Neurosci. 13:38. 10.3389/fnmol.2020.00038 32265650 PMC7096580

[B51] Jack C. R. Jr. Albert M. S. Knopman D. S. McKhann G. M. Sperling R. A. Carrillo M. C.. (2011). Introduction to the recommendations from the National Institute on Aging-Alzheimer's Association workgroups on diagnostic guidelines for Alzheimer's disease. Alzheimers Dement. 7, 257–262. 10.1016/j.jalz.2011.03.004 21514247 PMC3096735

[B52] Jay T. R. Hirsch A. M. Broihier M. L. Miller C. M. Neilson L. E. Ransohoff R. M.. (2017). Disease progression-dependent effects of TREM2 deficiency in a mouse model of Alzheimer's disease. J. Neurosci. 37, 637–647. 10.1523/JNEUROSCI.2110-16.2016 28100745 PMC5242410

[B53] Jia L. Qiu Q. Zhang H. Chu L. Du Y. Zhang J.. (2019). Concordance between the assessment of Aβ42, T-tau, and P-T181-tau in peripheral blood neuronal-derived exosomes and cerebrospinal fluid. Alzheimers Dement. 15, 1071–1080. 10.1016/j.jalz.2019.05.002 31422798

[B54] Jia L. Zhu M. Kong C. Pang Y. Zhang H. Qiu Q.. (2021). Blood neuro-exosomal synaptic proteins predict Alzheimer's disease at the asymptomatic stage. Alzheimers Dement. 17, 49–60. 10.1002/alz.12166 32776690 PMC7984076

[B55] Joshi P. Benussi L. Furlan R. Ghidoni R. Verderio C. (2015). Extracellular vesicles in Alzheimer's disease: friends or foes? Focus on aβ-vesicle interaction. Int. J. Mol. Sci. 16, 4800–4813. 10.3390/ijms16034800 25741766 PMC4394450

[B56] Joshi P. Turola E. Ruiz A. Bergami A. Libera D. D. Benussi L.. (2014). Microglia convert aggregated amyloid-β into neurotoxic forms through the shedding of microvesicles. Cell Death Differ. 21, 582–593. 10.1038/cdd.2013.180 24336048 PMC3950321

[B57] Kapogiannis D. Boxer A. Schwartz J. B. Abner E. L. Biragyn A. Masharani U.. (2015). Dysfunctionally phosphorylated type 1 insulin receptor substrate in neural-derived blood exosomes of preclinical Alzheimer's disease. FASEB J. 29, 589–596. 10.1096/fj.14-262048 25342129 PMC4314222

[B58] Keren-Shaul H. Spinrad A. Weiner A. Matcovitch-Natan O. Dvir-Szternfeld R. Ulland T. K.. (2017). A unique microglia type associated with restricting development of Alzheimer's disease. Cell 169, 1276–1290.e17. 10.1016/j.cell.2017.05.018 28602351

[B59] Khongkow M. Yata T. Boonrungsiman S. Ruktanonchai U. R. Graham D. Namdee K. (2019). Surface modification of gold nanoparticles with neuron-targeted exosome for enhanced blood-brain barrier penetration. Sci. Rep. 9:8278. 10.1038/s41598-019-44569-6 31164665 PMC6547645

[B60] Krämer-Albers E. M. Bretz N. Tenzer S. Winterstein C. Möbius W. Berger H.. (2007). Oligodendrocytes secrete exosomes containing major myelin and stress-protective proteins: trophic support for axons? Proteomics Clin. Appl. 1, 1446–1461. 10.1002/prca.200700522 21136642

[B61] Lai R. C. Arslan F. Lee M. M. Sze N. S. Choo A. Chen T. S.. (2010). Exosome secreted by MSC reduces myocardial ischemia/reperfusion injury. Stem Cell Res. 4, 214–222. 10.1016/j.scr.2009.12.003 20138817

[B62] Landeiro F. Walsh K. Ghinai I. Mughal S. Nye E. Wace H.. (2018). Measuring quality of life of people with predementia and dementia and their caregivers: a systematic review protocol. BMJ Open 8:e019082. 10.1136/bmjopen-2017-019082 29602838 PMC5884378

[B63] Liddelow S. A. Barres B. A. (2017). Reactive astrocytes: production, function, therapeutic potential. Immunity 46, 957–967. 10.1016/j.immuni.2017.06.006 28636962

[B64] Lim C. Z. J. Zhang Y. Chen Y. Zhao H. Stephenson M. C. Ho N. R. Y.. (2019). Subtyping of circulating exosome-bound amyloid β reflects brain plaque deposition. Nat. Commun. 10:1144. 10.1038/s41467-019-09030-2 30850633 PMC6408581

[B65] Lino M. M. Simões S. Tomatis F. Albino I. Barrera A. Vivien D.. (2021). Engineered extracellular vesicles as brain therapeutics. J. Control. Release 338, 472–485. 10.1016/j.jconrel.2021.08.037 34428481

[B66] Liu S. Fan M. Xu J. X. Yang L. J. Qi C. C. Xia Q. R.. (2022). Exosomes derived from bone-marrow mesenchymal stem cells alleviate cognitive decline in AD-like mice by improving BDNF-related neuropathology. J. Neuroinflammation 19:35. 10.1186/s12974-022-02393-2 35130907 PMC8822863

[B67] Longobardi A. Benussi L. Nicsanu R. Bellini S. Ferrari C. Saraceno C.. (2021). Plasma extracellular vesicle size and concentration are altered in Alzheimer's disease, dementia with Lewy bodies, frontotemporal dementia. Front. Cell Dev. Biol. 9:667369. 10.3389/fcell.2021.667369 34046409 PMC8148014

[B68] Mathews P. M. Levy E. (2019). Exosome production is key to neuronal endosomal pathway integrity in neurodegenerative diseases. Front. Neurosci. 13:1347. 10.3389/fnins.2019.01347 31911768 PMC6920185

[B69] Mrak R. E. Sheng J. G. Griffin W. S. (1996). Correlation of astrocytic S100 beta expression with dystrophic neurites in amyloid plaques of Alzheimer's disease. J. Neuropathol. Exp. Neurol. 55, 273–279. 10.1097/00005072-199603000-00002 8786385 PMC3833601

[B70] Muraoka S. Jedrychowski M. P. Tatebe H. DeLeo A. M. Ikezu S. Tokuda T.. (2019). Proteomic profiling of extracellular vesicles isolated from cerebrospinal fluid of former national football league players at risk for chronic traumatic encephalopathy. Front. Neurosci. 13:1059. 10.3389/fnins.2019.01059 31649498 PMC6794346

[B71] Muraoka S. Jedrychowski M. P. Yanamandra K. Ikezu S. Gygi S. P. Ikezu T. (2020). Proteomic profiling of extracellular vesicles derived from cerebrospinal fluid of Alzheimer's disease patients: a pilot study. Cells 9:1959. 10.3390/cells9091959 32854315 PMC7565882

[B72] Murgoci A. N. Cizkova D. Majerova P. Petrovova E. Medvecky L. Fournier I.. (2018). Brain-cortex microglia-derived exosomes: nanoparticles for glioma therapy. Chemphyschem 19, 1205–1214. 10.1002/cphc.201701198 29327816

[B73] Nassar W. El-Ansary M. Sabry D. Mostafa M. A. Fayad T. Kotb E.. (2016). Umbilical cord mesenchymal stem cells derived extracellular vesicles can safely ameliorate the progression of chronic kidney diseases. Biomater. Res. 20:21. 10.1186/s40824-016-0068-0 27499886 PMC4974791

[B74] Neumann H. Kotter M. R. Franklin R. J. (2009). Debris clearance by microglia: an essential link between degeneration and regeneration. Brain 132, 288–295. 10.1093/brain/awn109 18567623 PMC2640215

[B75] Nogueras-Ortiz C. J. Mahairaki V. Delgado-Peraza F. Das D. Avgerinos K. Eren E.. (2020). Astrocyte- and Neuron-derived extracellular vesicles from Alzheimer's disease patients effect complement-mediated neurotoxicity. Cells 9:1618. 10.3390/cells9071618 32635578 PMC7407141

[B76] Pan J. He R. Huo Q. Shi Y. Zhao L. (2020). Brain Microvascular endothelial cell derived exosomes potently ameliorate cognitive dysfunction by enhancing the clearance of Aβ through up-regulation of P-gp in mouse model of AD. Neurochem. Res. 45, 2161–2172. 10.1007/s11064-020-03076-1 32583212

[B77] Paolicelli R. C. Bergamini G. Rajendran L. (2019). Cell-to-cell communication by extracellular vesicles: focus on microglia. Neuroscience 405, 148–157. 10.1016/j.neuroscience.2018.04.003 29660443

[B78] Peng K. Y. Pérez-González R. Alldred M. J. Goulbourne C. N. Morales-Corraliza J. Saito M.. (2019). Apolipoprotein E4 genotype compromises brain exosome production. Brain 142, 163–175. 10.1093/brain/awy289 30496349 PMC6308312

[B79] Prince M. Bryce R. Albanese E. Wimo A. Ribeiro W. Ferri C. P. (2013). The global prevalence of dementia: a systematic review and metaanalysis. Alzheimers Dement. 9, 63–75.e2. 10.1016/j.jalz.2012.11.007 23305823

[B80] Proia P. Schiera G. Mineo M. Ingrassia A. M. Santoro G. G. Savettieri G.. (2008). Astrocytes shed extracellular vesicles that contain fibroblast growth factor-2 and vascular endothelial growth factor. Int. J. Mol. Med. 21, 63–67. 10.3892/ijmm.21.1.63 18097617

[B81] Pulliam L. Sun B. Mustapic M. Chawla S. Kapogiannis D. (2019). Plasma neuronal exosomes serve as biomarkers of cognitive impairment in HIV infection and Alzheimer's disease. J. Neurovirol. 25, 702–709. 10.1007/s13365-018-0695-4 30610738 PMC7372698

[B82] Rani K. Rastogi S. Vishwakarma P. Bharti P. S. Sharma V. Renu K.. (2021). A novel approach to correlate the salivary exosomes and their protein cargo in the progression of cognitive impairment into Alzheimer's disease. J. Neurosci. Methods 347:108980. 10.1016/j.jneumeth.2020.108980 33075328

[B83] Ratajczak J. Miekus K. Kucia M. Zhang J. Reca R. Dvorak P.. (2006). Embryonic stem cell-derived microvesicles reprogram hematopoietic progenitors: evidence for horizontal transfer of mRNA and protein delivery. Leukemia 20, 847–856. 10.1038/sj.leu.2404132 16453000

[B84] Riancho J. Vázquez-Higuera J. L. Pozueta A. Lage C. Kazimierczak M. Bravo M.. (2017). MicroRNA profile in patients with Alzheimer's disease: analysis of miR-9-5p and miR-598 in raw and exosome enriched cerebrospinal fluid samples. J. Alzheimers Dis. 57, 483–491. 10.3233/JAD-161179 28269782

[B85] Rosas-Hernandez H. Cuevas E. Raymick J. B. Robinson B. L. Ali S. F. Hanig J.. (2019). Characterization of serum exosomes from a transgenic mouse model of Alzheimer's disease. Curr. Alzheimer Res. 16, 388–395. 10.2174/1567205016666190321155422 30907317

[B86] Ruan Z. Delpech J. C. Venkatesan Kalavai S. Van Enoo A. A. Hu J. Ikezu S.. (2020). P2RX7 inhibitor suppresses exosome secretion and disease phenotype in P301S tau transgenic mice. Mol. Neurodegener. 15:47. 10.1186/s13024-020-00396-2 32811520 PMC7436984

[B87] Ruan Z. Pathak D. Venkatesan Kalavai S. Yoshii-Kitahara A. Muraoka S. Bhatt N.. (2021). Alzheimer's disease brain-derived extracellular vesicles spread tau pathology in interneurons. Brain. 144, 288–309. 10.1093/brain/awaa376 33246331 PMC7880668

[B88] Saugstad J. A. Lusardi T. A. Van Keuren-Jensen K. R. Phillips J. I. Lind B. Harrington C. A.. (2017). Analysis of extracellular RNA in cerebrospinal fluid. J. Extracell. Vesicles 6:1317577. 10.1080/20013078.2017.1317577 28717417 PMC5505019

[B89] Sbai O. Ould-Yahoui A. Ferhat L. Gueye Y. Bernard A. Charrat E.. (2010). Differential vesicular distribution and trafficking of MMP-2, MMP-9, and their inhibitors in astrocytes. Glia 58, 344–366. 10.1002/glia.20927 19780201

[B90] Schafer D. P. Lehrman E. K. Kautzman A. G. Koyama R. Mardinly A. R. Yamasaki R.. (2012). Microglia sculpt postnatal neural circuits in an activity and complement-dependent manner. Neuron 74, 691–705. 10.1016/j.neuron.2012.03.026 22632727 PMC3528177

[B91] Sivandzade F. Cucullo L. (2021). Regenerative stem cell therapy for neurodegenerative diseases: an overview. Int. J. Mol. Sci. 22:2153. 10.3390/ijms22042153 33671500 PMC7926761

[B92] Söllvander S. Nikitidou E. Brolin R. Söderberg L. Sehlin D. Lannfelt L.. (2016). Accumulation of amyloid-β by astrocytes result in enlarged endosomes and microvesicle-induced apoptosis of neurons. Mol. Neurodegener. 11:38. 10.1186/s13024-016-0098-z 27176225 PMC4865996

[B93] Song Z. Qu Y. Xu Y. Zhang L. Zhou L. Han Y.. (2021). Microarray microRNA profiling of urinary exosomes in a 5XFAD mouse model of Alzheimer's disease. Anim. Model Exp. Med. 4, 233–242. 10.1002/ame2.12175 34557649 PMC8446702

[B94] Song Z. Xu Y. Deng W. Zhang L. Zhu H. Yu P.. (2020a). Brain derived exosomes are a double-edged sword in Alzheimer's disease. Front. Mol. Neurosci. 13:79. 10.3389/fnmol.2020.00079 32547364 PMC7274346

[B95] Song Z. Xu Y. Zhang L. Zhou L. Zhang Y. Han Y.. (2020b). Comprehensive proteomic profiling of urinary exosomes and identification of potential non-invasive early biomarkers of Alzheimer's disease in 5XFAD mouse model. Front. Genet. 11:565479. 10.3389/fgene.2020.565479 33250918 PMC7674956

[B96] Street J. M. Barran P. E. Mackay C. L. Weidt S. Balmforth C. Walsh T. S.. (2012). Identification and proteomic profiling of exosomes in human cerebrospinal fluid. J. Transl. Med. 10:5. 10.1186/1479-5876-10-5 22221959 PMC3275480

[B97] Sun R. Wang H. Shi Y. Gao D. Sun Z. Chen Z.. (2019). A pilot study of urinary exosomes in Alzheimer's disease. Neurodegener. Dis. 19, 184–191. 10.1159/000505851 32375155

[B98] Sun R. Wang H. Shi Y. Sun Z. Jiang H. Zhang J. (2020). Changes in the morphology, number, and pathological protein levels of plasma exosomes may help diagnose Alzheimer's disease. J. Alzheimers Dis. 73, 909–917. 10.3233/JAD-190497 31884461

[B99] Szatanek R. Baj-Krzyworzeka M. Zimoch J. Lekka M. Siedlar M. Baran J. (2017). The methods of choice for Extracellular Vesicles (EVs) characterization. Int. J. Mol. Sci. 18:1153. 10.3390/ijms18061153 28555055 PMC5485977

[B100] Tamboli I. Y. Barth E. Christian L. Siepmann M. Kumar S. Singh S.. (2010). Statins promote the degradation of extracellular amyloid {beta}-peptide by microglia via stimulation of exosome-associated insulin-degrading enzyme (IDE) secretion. J. Biol. Chem. 285, 37405–37414. 10.1074/jbc.M110.149468 20876579 PMC2988346

[B101] Ter-Ovanesyan D. Norman M. Lazarovits R. Trieu W. Lee J. H. Church G.. (2021). Framework for rapid comparison of extracellular vesicle isolation methods. Elife 10:e70725. 10.7554/eLife.70725.sa2 34783650 PMC8651285

[B102] Upadhya R. Zingg W. Shetty S. Shetty A. K. (2020). Astrocyte-derived extracellular vesicles: Neuroreparative properties and role in the pathogenesis of neurodegenerative disorders. J. Control Release 323, 225–239. 10.1016/j.jconrel.2020.04.017 32289328 PMC7299747

[B103] van der Pol E. Böing A. N. Harrison P. Sturk A. Nieuwland R. (2012). Classification, functions, and clinical relevance of extracellular vesicles. Pharmacol. Rev. 64, 676–705. 10.1124/pr.112.005983 22722893

[B104] Vasic V. Barth K. Schmidt M. H. H. (2019). Neurodegeneration and Neuro-Regeneration-Alzheimer's disease and stem cell therapy. Int. J. Mol. Sci. 20:4272. 10.3390/ijms20174272 31480448 PMC6747457

[B105] Venugopal C. Shamir C. Senthilkumar S. Babu J. V. Sonu P. K. Nishtha K. J.. (2017). Dosage and passage dependent neuroprotective effects of exosomes derived from rat bone marrow mesenchymal stem cells: an *in vitro* analysis. Curr. Gene Ther. 17, 379–390. 10.2174/1566523218666180125091952 29366415

[B106] Villemagne V. L. Burnham S. Bourgeat P. Brown B. Ellis K. A. Salvado O.. (2013). Amyloid β deposition, neurodegeneration, and cognitive decline in sporadic Alzheimer's disease: a prospective cohort study. Lancet Neurol. 12, 357–367. 10.1016/S1474-4422(13)70044-9 23477989

[B107] Wang S. Cesca F. Loers G. Schweizer M. Buck F. Benfenati F.. (2011). Synapsin I is an oligomannose-carrying glycoprotein, acts as an oligomannose-binding lectin, and promotes neurite outgrowth and neuronal survival when released via glia-derived exosomes. J. Neurosci. 31, 7275–7290. 10.1523/JNEUROSCI.6476-10.2011 21593312 PMC6622588

[B108] Watson L. S. Hamlett E. D. Stone T. D. Sims-Robinson C. (2019). Neuronally derived extracellular vesicles: an emerging tool for understanding Alzheimer's disease. Mol. Neurodegener. 14:22. 10.1186/s13024-019-0317-5 31182115 PMC6558712

[B109] Wei H. Xu Y. Chen Q. Chen H. Zhu X. Li Y. (2020). Mesenchymal stem cell-derived exosomal miR-223 regulates neuronal cell apoptosis. Cell Death Dis. 11:290. 10.1038/s41419-020-2490-4 32341353 PMC7184756

[B110] Winston C. N. Goetzl E. J. Akers J. C. Carter B. S. Rockenstein E. M. Galasko D.. (2016). Prediction of conversion from mild cognitive impairment to dementia with neuronally derived blood exosome protein profile. Alzheimers Dement. 3, 63–72. 10.1016/j.dadm.2016.04.001 27408937 PMC4925777

[B111] Winston C. N. Goetzl E. J. Schwartz J. B. Elahi F. M. Rissman R. A. (2019). Complement protein levels in plasma astrocyte-derived exosomes are abnormal in conversion from mild cognitive impairment to Alzheimer's disease dementia. Alzheimers Dement. 11, 61–66. 10.1016/j.dadm.2018.11.002 31032394 PMC6477776

[B112] Wyss-Coray T. Loike J. D. Brionne T. C. Lu E. Anankov R. Yan F.. (2003). Adult mouse astrocytes degrade amyloid-beta *in vitro* and *in situ*. Nat. Med. 9, 453–457. 10.1038/nm838 12612547

[B113] Xie H. M. Su X. Zhang F. Y. Dai C. L. Wu R. H. Li Y.. (2022). Profile of the RNA in exosomes from astrocytes and microglia using deep sequencing: implications for neurodegeneration mechanisms. Neural Regen. Res. 17, 608–617. 10.4103/1673-5374.320999 34380901 PMC8504369

[B114] Xing W. Gao W. Lv X. Xu X. Zhang Z. Yan J.. (2021). The diagnostic value of exosome-derived biomarkers in Alzheimer's disease and mild cognitive impairment: a meta-analysis. Front. Aging Neurosci. 13:637218. 10.3389/fnagi.2021.637218 33732139 PMC7957006

[B115] Yin Z. Han Z. Hu T. Zhang S. Ge X. Huang S.. (2020). Neuron-derived exosomes with high miR-21-5p expression promoted polarization of M1 microglia in culture. Brain Behav. Immun. 83, 270–282. 10.1016/j.bbi.2019.11.004 31707083

[B116] Yokoi A. Villar-Prados A. Oliphint P. A. Zhang J. Song X. De Hoff P.. (2019). Mechanisms of nuclear content loading to exosomes. Sci. Adv. 5:eaax8849. 10.1126/sciadv.aax8849 31799396 PMC6867874

[B117] Yokose T. Kabe Y. Matsuda A. Kitago M. Matsuda S. Hirai M.. (2020). O-Glycan-Altered extracellular vesicles: a specific serum marker elevated in pancreatic cancer. Cancers 12:2469. 10.3390/cancers12092469 32878320 PMC7563872

[B118] Yu Z. Shi M. Stewart T. Fernagut P. O. Huang Y. Tian C.. (2020). Reduced oligodendrocyte exosome secretion in multiple system atrophy involves SNARE dysfunction. Brain 143, 1780–1797. 10.1093/brain/awaa110 32428221 PMC7296853

[B119] Yuyama K. Sun H. Mitsutake S. Igarashi Y. (2012). Sphingolipid-modulated exosome secretion promotes clearance of amyloid-β by microglia. J. Biol. Chem. 287, 10977–10989. 10.1074/jbc.M111.324616 22303002 PMC3322859

[B120] Yuyama K. Sun H. Sakai S. Mitsutake S. Okada M. Tahara H.. (2014). Decreased amyloid-β pathologies by intracerebral loading of glycosphingolipid-enriched exosomes in Alzheimer model mice. J. Biol. Chem. 289, 24488–24498. 10.1074/jbc.M114.577213 25037226 PMC4148874

[B121] Yuyama K. Sun H. Usuki S. Sakai S. Hanamatsu H. Mioka T.. (2015). A potential function for neuronal exosomes: sequestering intracerebral amyloid-β peptide. FEBS Lett. 589, 84–88. 10.1016/j.febslet.2014.11.027 25436414

[B122] Yuyama K. Takahashi K. Usuki S. Mikami D. Sun H. Hanamatsu H.. (2019). Plant sphingolipids promote extracellular vesicle release and alleviate amyloid-β pathologies in a mouse model of Alzheimer's disease. Sci. Rep. 9:16827. 10.1038/s41598-019-53394-w 31727994 PMC6856149

[B123] Zhai L. Shen H. Sheng Y. Guan Q. (2021). ADMSC Exo-MicroRNA-22 improve neurological function and neuroinflammation in mice with Alzheimer's disease. J. Cell. Mol. Med. 25, 7513–7523. 10.1111/jcmm.16787 34250722 PMC8335682

[B124] Zhang J. Buller B. A. Zhang Z. G. Zhang Y. Lu M. Rosene D. L.. (2021). Exosomes derived from bone marrow mesenchymal stromal cells promote remyelination and reduce neuroinflammation in the demyelinating central nervous system. Exp. Neurol. 347:113895. 10.1016/j.expneurol.2021.113895 34653510 PMC12050987

[B125] Zhao J. O'Connor T. Vassar R. (2011). The contribution of activated astrocytes to Aβ production: implications for Alzheimer's disease pathogenesis. J. Neuroinflammation 8:150. 10.1186/1742-2094-8-150 22047170 PMC3216000

[B126] Zheng J. Yan H. Shi L. Kong Y. Zhao Y. Xie L.. (2016). The CYP19A1 rs3751592 variant confers susceptibility to Alzheimer disease in the Chinese Han population. Medicine 95:e4742. 10.1097/MD.0000000000004742 27583919 PMC5008603

